# Temporal assessment of water and soil quality near Barapukuria coal mine, Bangladesh

**DOI:** 10.1016/j.heliyon.2024.e40722

**Published:** 2024-11-27

**Authors:** Md Asif All Azad, Abu Bakker Chiddiq, Md Rubel Miah, Md Hafijur Rahman Sabbir

**Affiliations:** Department of Chemical Engineering, Khulna University of Engineering & Technology (KUET), Khulna, 9203, Bangladesh

**Keywords:** Barapukuria coal mine, Seasonal variations, Water quality index, Geo-accumulation index, Heavy metal contamination, Soil fertility

## Abstract

This study assessed the water suitability for various purposes, evaluated heavy metal contamination and soil fertility, and investigated seasonal variations in water and soil parameters near the Barapukuria coal mine in Bangladesh. A total of nine sampling locations were selected, resulting in 18 samples (12 water and 6 soil) collected during the summer and winter seasons. The water samples were analyzed at the Environmental Engineering Laboratory, while the soil samples were analyzed at the Soil Resource Development Institute (SRDI). The suitability of drinking water was evaluated using the Water Quality Index (WQI), and heavy metal contamination was assessed using the geo-accumulation index. The findings indicate that surface water is generally suitable for irrigation due to several positive attributes, with slight to moderate seasonal variations. However, treatment is required for high turbidity and chemical oxygen demand (COD). On the other hand, wastewater quality exhibits elevated levels of turbidity, total suspended solids, and chemical oxygen demand, although most parameters are within acceptable limits. The assessment of drinking water quality using the WQI reveals that both hand and deep tubewells are generally unfit for consumption. The WQI values peak at 441.84 in summer and 202.79 in winter for the hand tubewell, and 129.66 in summer and 95.88 in winter for the deep tubewell. This finding emphasizes the need for effective treatment and continuous monitoring. The soil analysis indicates that most heavy metals are not present in significant amounts, except for cadmium, which shows uncontaminated to moderately contaminated conditions during the summer. Moreover, the soil treated with coal water exhibits significantly higher levels of organic matter, micronutrients, and macronutrients compared to the normal field soil. Further analysis of the coal water treated soil shows a shift from a slightly acidic pH in summer (6.30) to a neutral pH in winter (7.10). The organic matter content remains consistently high, and nutrient analysis shows high to very high levels of Zn, Cu, K, Ca, Mg, Mn, and Fe, while phosphorus remains low. Seasonal analysis reveals increased levels of zinc, potassium, and phosphorus in winter, whereas other nutrients generally decrease. The primary novel contribution of this study is the detailed seasonal analysis of water and soil quality near the coal mining area, capturing changes across summer and winter. Additionally, it compares coal water treated soil with normal field soil, revealing significant improvements in soil quality and fertility.

**Table:** Abbreviations

BCM	Barapukuria Coal Mine
WHO	World Health Organization
WQI	Water Quality Index
I_geo_	Geo-accumulation Index
SRDI	Soil Resource Development Institute
BARC	Bangladesh Agricultural Research Council
EC	Electrical Conductivity
COD	Chemical Oxygen Demand
BOD	Biochemical Oxygen Demand
TS	Total Solids
TDS	Total Dissolved Solids
TSS	Total Suspended Solids
DO	Dissolved Oxygen
ECR	Environmental Conservation Rule
TC	Total Coliform
FC	Fecal Coliform
DTPA	Diethylene-Triamine-Penta Acetic Acid
EBT	Erichrome Black T

## 1 Introduction

Water is a vital natural resource extensively used for industrial, commercial, domestic, and agricultural purposes worldwide. Despite its abundance, maintaining water quality is a significant challenge due to the numerous sources of discharge into the environment. Regions with mining and mineral processing industries face more severe water quality issues, often resulting in contamination that affects ecosystems and human health [1]. Mining operations often generate various waste streams that can contribute to different types of pollution, ultimately resulting in contamination [2]. Therefore, it is crucial to assess groundwater resources in developing countries, such as Bangladesh. In this country, groundwater sources provide 70 % of irrigation water and 95 % of drinking water in both rural and urban areas [3]. Furthermore, agriculture plays a vital role in the economic growth of developing nations like Bangladesh, and soil quality is essential for agricultural productivity [4]. However, industrialization has had detrimental impacts on agriculture globally. Mining, in particular, poses significant environmental risks. Additionally, mining waste contributes to substantial soil degradation [5].

Coal mining poses a significant threat to the ecological integrity of mining regions. It can result in soil degradation, surface subsidence, vegetation loss, ecosystem disruption, biodiversity decline, landscape alteration, and reduced crop yields [6]. The excessive release of carbon into the atmosphere hinders plants' capacity to capture and store carbon. While numerous studies delve into the mechanisms of carbon release, research on the environmental consequences of coal mining remains relatively limited. Nevertheless, coal mining is a primary contributor to anthropogenic greenhouse gas emissions, releasing substantial amounts of carbon into the atmosphere. Acid mine drainage (AMD) is a significant environmental problem in coal mining regions with high sulfur content. It occurs when sulfide minerals react with oxygen and water, producing sulfuric acid [7]. This acidic effluent leaches heavy metals into nearby water bodies, causing contamination. Fly ash, a byproduct of coal combustion, can effectively neutralize AMD due to its substantial alkalinity [8]. Mine drainage contains elevated levels of metals and metalloids, contaminating groundwater and soil. This can be a primary source of environmental pollution [9]. The discharge of mining waste into the environment can cause severe ecological devastation, hindering complete restoration or rehabilitation of affected areas. Coal mine drainage exhibits variable composition, ranging from acidic to alkaline, and often contains high concentrations of heavy metals such as nickel (Ni), copper (Cu), manganese (Mn), and iron (Fe). When dissolved, these metals can corrode infrastructure, pose toxicity risks, cause encrustation, and severely impact aquatic ecosystems and water supplies [10]. Certain heavy metals, such as copper, nickel, iron, and zinc, are essential micronutrients for plants and animals. However, both excessive and deficient levels of these elements can lead to various health problems.

Coal is a brownish-black or black sedimentary rock formed from the compressed remains of ancient vegetation over approximately 100–400 million years [11]. Coal is composed of a complex mixture of carbon, hydrogen, oxygen, nitrogen, sulfur, and mineral matter. The Barapukuria coal mine is classified as a red category industry [12]. The primary constituents of Barapukuria coal are as follows: 12.4 % ash, 0.53 % sulfur, and 10 % moisture [13]. The Barapukuria coal mine produces bituminous coal, a highly volatile type with a high calorific value. As the first in Bangladesh to adopt underground coal mining, the mine initiated multi-slice longwall operations in 2005. By the end of the 2017–2018 fiscal year, a cumulative total of 923,276.080 metric tons of coal had been extracted [14]. The mining operation discharges untreated underground sump water to the surface. Additionally, inadequate coal storage infrastructure allows for the easy dispersion of coal dust into surrounding soil, contaminating it and adversely affecting the vast paddy fields in the Barapukuria coal mine region [15].

Soil is the primary reservoir for plant nutrients, supplying essential elements vital for plant growth and development [16]. Nutrient availability, particularly micronutrients, is critical for sustainable agricultural productivity. Micronutrient deficiencies hinder soil health, stability, and crop yields. Both macronutrients and micronutrients are essential for optimal plant growth. While required in smaller quantities, micronutrients are equally vital as macronutrients in supporting plant development, soil fertility, and animal health. Key plant nutrients include magnesium, phosphorus, potassium, nitrogen, sulfur, calcium, iron, copper, and manganese [17]. Compared to many developed countries, Bangladesh faces significantly lower crop yields [18]. Several factors contribute to Bangladesh's low crop yields, with soil health being a primary challenge. Soil toxicity, nutrient deficiencies, poor crop and soil management, land-use changes, pest and disease outbreaks, and natural disasters pose significant obstacles to agricultural productivity [19]. Additionally, mining activities significantly impact the region's agricultural lands and water bodies through the discharge of untreated underground sump water and the dispersion of coal dust. The cumulative effects of mining operations pose substantial threats to both terrestrial and aquatic ecosystems [20]. The Barapukuria mining activities have affected about 2500 residents in seven villages and have led to the destruction of nearly 300 acres of land, as reported by the International Accountability Project [21]. Land subsidence deeper than 1 m has resulted in the destruction of crops, lands, and homes. Moreover, the substantial water withdrawals from the Barapukuria mine have deprived residents of 15 villages of access to water [22].

Nguyen et al. [23] conducted a statistical analysis of groundwater quality in Ben Tre Province, Vietnam, revealing significant seasonal variations. Total dissolved solids (TDS), salinity, and coliform levels were markedly higher during the summer season compared to the winter season, indicating a decline in groundwater quality. The study identified TDS, salinity, E. coli, and coliforms as predominant contaminants. Similarly, Shirin et al. [24] carried out a two-year study (2010–2011) assessing the physicochemical parameters of municipal wastewater discharged into the Ganga River in Haridwar, India. Monthly monitoring of key parameters revealed that wastewater quality generally complied with regulatory standards. Nguyen et al. [25] also assessed surface water quality in Soc Trang, Vietnam, identifying contamination by organic matter, nutrients, microorganisms, salinity, and iron. The WQI fluctuated seasonally, with consistently high TSS, COD, iron, and coliform levels indicating persistent pollution. This study underscores the importance of seasonal monitoring for effective water quality management.

While the existing literature has provided valuable insights into the environmental impacts of the Barapukuria coal mine, there are still several important areas that need further exploration. Specifically, there is a need for more detailed analysis of water and soil quality throughout different seasons, as well as assessments of soil fertility and agricultural suitability. Therefore, the main objectives of this study are as follows.

(i) To evaluate the suitability of water for irrigation, disposal, and drinking purposes
(ii) To measure and assess the extent of heavy metal contamination, as well as evaluate soil fertility
(iii) To investigate how water and soil parameters vary across different seasons

## 2 Materials and methods

### 2.1 Study area

**Fig. 1:**
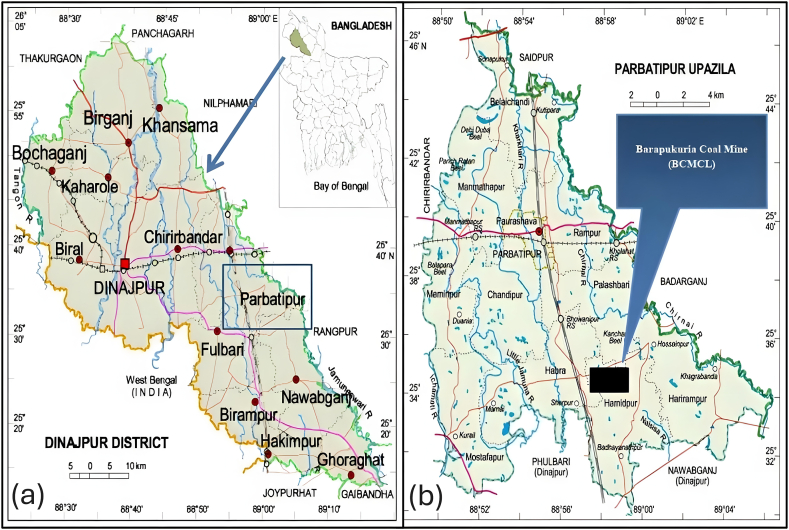
Location of the study area - Barapukuria Coal Mine, Bangladesh. The map illustrates the geographical location of the Barapukuria Coal Mine within the Parbatipur Upazila of Dinajpur District, Bangladesh. Panel (a) shows the position of Parbatipur within Dinajpur District, while panel (b) provides a detailed view of Parbatipur Upazila, highlighting the specific location of the Barapukuria Coal Mine.

The Barapukuria coal mine and power station are located in northwestern Bangladesh, around 45 km east of Dinajpur, the district capital, and 20 km from the Indian border. The site is part of the Dinajpur Shield, which is bounded by the Indian Peninsular Shield to the west, the Shillong Shield to the east, and the Himalayan foredeep to the north. The verified coal reserve officially covers an area of 5.25 square kilometers, with the potential for expansion by 1–1.5 square kilometers to the south [26]. The power plant and mine are administratively situated within the Hamidpur union of Parbatipur Upazila, Dinajpur district. Geographically, their coordinates range from 25°31′45″ to 25°33′05″ N in latitude and from 88°57′48″ to 88°58′53″ E in longitude [27]. The coalfield includes several villages, including Barapukuria, Banspukur, Dakshin Rasulpur, Kalupara, Hamidpur, Chauhati, Ichabpur, Patigram, Gopalpara, Baidyanathpur, Sherpur, and Baigram [28]. Fig. 1 illustrates the geographical context of the study area.

### 2.2 Research planning and challenges

The research planning for the study involved meticulous strategizing to ensure representative sampling. Surface water samples were collected from areas adjacent to irrigation lands, and the utilization of this water for irrigation was corroborated through preliminary surveys conducted with local residents. Drinking water samples were obtained from the local market near the mining area, specifically targeting water intended for human consumption, thus allowing an assessment of the water quality consumed by the local population. Wastewater samples were collected from points where coal mining operations released water to assess its suitability for disposal into the environment. After consulting with local residents to confirm that the land was being irrigated with surface water, a soil sample was collected. Additionally, a field soil sample irrigated with groundwater was obtained for comparative analysis. A surface soil sample near mine drainage was also collected to check for any heavy metal contamination. All samples were collected within a 1-km radius of the mining site. Initially, standard values were derived from reputable sources such as the Environmental Conservation Rule (ECR, 2023), Surface Water Quality Standards (IS: 2296), and relevant scientific literature, including general standards for effluent discharge and peer-reviewed research publications, guidelines from IS: 10500 for drinking water quality, and the Bangladesh Agricultural Research Council (BARC) for soil. These sources provided a comprehensive list of parameters required for assessing the quality of irrigation water, suitability of wastewater disposal, drinking water quality, heavy metal contamination in surface soil near mine drainage, and soil suitability for irrigation. Various parameters were enlisted for the assessment of irrigation water quality, environmental suitability of wastewater disposal, drinking water quality, heavy metal contamination in surface soil near mine drainage, and soil suitability for irrigation. The final selection of parameters was based on the availability of testing facilities in the Environmental Engineering Laboratory for water samples and the Soil Resource Development Institute (SRDI) for soil samples. Furthermore, certain parameters that had not been previously evaluated or were not assessed seasonally were included in this study due to their significance.

This study encountered various challenges in capturing and analyzing seasonal variations in water and soil quality parameters. The planning for sample collection during both summer and winter posed logistical difficulties. The lack of comprehensive seasonal data in previous studies conducted in the Barapukuria coal mining area required a novel approach to seasonal analysis, which complicated baseline comparisons. The study uniquely compares coal water treated soil with normal field soil, demonstrating significant improvements in soil quality and fertility resulting from coal water treatment. By identifying specific parameters that exceed guidelines and their seasonal variations, the study provides vital information for developing targeted environmental management strategies in mining areas. In contrast to previous publications that provided a snapshot of environmental conditions, this study incorporates detailed seasonal analysis, providing insights into temporal variations and their implications. Furthermore, the inclusion of new, seasonally adjusted data sets expands the scope of environmental analysis, enhancing our understanding of the impacts of coal mining activities over time.

### 2.3 Selection of sampling locations

**Fig. 2:**
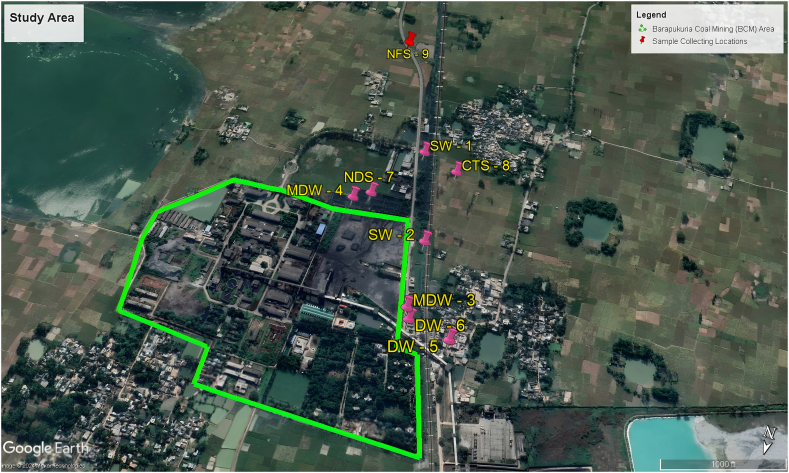
Location of water and soil sampling sites in the study area. The map, based on Google Earth imagery, displays the Barapukuria Coal Mine area, marked with a green boundary, and the specific locations of water and soil sampling sites (SW, MDW, NDS, CTS, NFS, DW) within and around the study area. The sampling points are indicated with pin markers, providing a clear overview of the sites where samples were collected for analysis.

Nine sampling locations were meticulously selected following an extensive reconnaissance survey. These locations encompassed two samples of surface water (pond water), two samples of wastewater, two samples of drinking water, one sample of surface soil in close proximity to the mine drainage, one sample of agricultural land irrigated with surface water near the mine drainage, and one sample of regular field soil irrigated with groundwater. The precise areas where the samples were collected are depicted in Fig. 2, while Table 1 provides the latitude, longitude, and distance from the mine site for each sampling location. To ascertain the precise locations of sample collection, a Garmin GPS 72H was utilized.

**Table 1:** Sampling locations and distances from the mine site.

Location	Latitude	Longitude	Distance from the mine site (m)
SW-1	25°32′35.67″N	88°57′25.11″E	504
SW-2	25°32′45.72″N	88°57′21.74″E	468
MDW-3	25°32′52.65″N	88°57′21.22″E	514
MDW-4	25°32′43.04″N	88°57′30.68″E	245
DW-5	25°32′54.47″N	88°57′16.38″E	660
DW-6	25°32′53.68″N	88°57′20.66″E	540
NDS-7	25°32′42″N	88°57′29″E	303
CTS-8	25°32′37″N	88°57′21″E	572
NFS-9	25°32′21″N	88°57′32″E	816

### 2.4 Collection of water samples

The timing of sample collection was carefully chosen to account for environmental factors. The samples were collected in the afternoon during both the summer season (May 2023) and the winter season (December 2023). Six 1.5-liter bottles were used to collect the water samples. Prior to sample collection, the bottles were washed three times with distilled water and thoroughly dried. The water samples were then transported in an insulated ice box equipped with ice packs to maintain a temperature between 2 and 8 °C throughout the 10-h journey to the laboratory. This method effectively preserves the integrity of the samples by ensuring a consistent low temperature. Upon arrival, the water samples were immediately placed in a refrigerator set at 4 °C in the Environmental Engineering Laboratory of Khulna University of Engineering & Technology (KUET) until analysis.

### 2.5 Water sample analysis

#### 2.5.1 Analytical methods

A comprehensive laboratory analysis was conducted to assess water quality near the Barapukuria coal mine in Bangladesh. The analysis aimed to ensure data accuracy and appropriate parameter evaluation by utilizing a range of sophisticated instruments and methodologies. The following methods were employed in this study to assess various water quality parameters:

**pH:** The pH of water samples was determined using a calibrated pH meter. The procedure involves immersing the pH meter's electrode in the water sample, ensuring thorough mixing to obtain a stable reading.

**Turbidity:** Turbidity measurements were obtained using a turbidity meter.

**Color:** The color of the water samples was assessed through spectrophotometric analysis using a DR-3900 spectrophotometer. This method involves measuring the absorbance of specific wavelengths of light by the water sample.

**Hardness:** A burette was filled with a standard ethylenediaminetetraacetic acid (EDTA) solution, ensuring the initial level was set to zero. A 50 mL aliquot of the water sample was then measured into a flask. Subsequently, 1 mL of ammonia buffer was added to the sample to maintain the pH level suitable for the indicator. Following this, 5 to 6 drops of Eriochrome Black T (EBT) indicator were introduced, resulting in the solution turning a wine-red color, indicating the presence of calcium and magnesium ions. The initial reading of the burette was recorded before commencing the titration. The sample was then titrated with the EDTA solution, with the titrant being added gradually while continuously swirling the flask. The titration was carried out until a distinct color change from wine-red to blue was observed, signifying the endpoint where all calcium and magnesium ions had been complexed by the EDTA. The volume of EDTA used at this endpoint was noted, and this value was utilized to calculate the total hardness of the water sample.

**Total Solids (TS):** To measure Total Solids (TS), a 100 mL aliquot of the thoroughly homogenized water sample was placed in aluminum weigh pans. The sample was evaporated on a hot plate and dried in a drying oven at 105 °C until a constant weight was achieved. The mass of the dried residue was then recorded. The formula used for calculating TS is shown in Equation (1):

(1) $$\text{TS}=\left(\frac{\;\text{Mass}\;\text{of}\;\text{dried}\;\text{sample}\;\text{at}\;105^{\circ }C-\text{Mass}\;\text{of}\;\text{weigh}\;\text{pan}\;}{\;\text{Volume}\;\text{of}\;\text{sample}\;}\right)\times 1000\frac{mg}{g}\times 1000\frac{mL}{L}$$

**Total Dissolved Solids (TDS):** To measure Total Dissolved Solids (TDS), a 50 mL water sample was first filtered through a filter paper to remove suspended solids. The filtrate, which contains the dissolved solids, was collected in a pre-weighed aluminum dish. This filtrate was then evaporated to dryness on a hot plate. After evaporation, the residue was dried in a drying oven at 105 °C to ensure complete removal of moisture. The dish was then cooled in a desiccator and reweighed to determine the mass of the dissolved solids. The formula used for calculating TDS is shown in Equation (2):

(2) $$\text{TDS}=\left(\frac{\;\text{Mass}\;\text{of}\;\text{dried}\;\text{sample}\;\text{residue}-\text{Mass}\;\text{of}\;\text{empty}\;\text{weigh}\;\text{pan}\;}{\;\text{Volume}\;\text{of}\;\text{sample}\;}\right)\times 1000\frac{mg}{g}\times 1000\frac{mL}{L}$$

**Total Suspended Solids (TSS):** The Total Suspended Solids (TSS) were calculated by subtracting the Total Dissolved Solids (TDS) from the Total Solids (TS). This indirect method ensures that all suspended particles are accounted for. The formula used for calculating TSS is as follows:

TSS=TS–TDS

**Electrical Conductivity (EC):** The electrical conductivity (EC) of the water samples was measured using a calibrated multimeter (Model WA-2015) at a standard temperature of 25 °C during the summer and 21 °C during the winter season.

**Dissolved Oxygen (DO):** The concentration of dissolved oxygen (DO) in water samples was measured using a calibrated DO meter.

**Biochemical Oxygen Demand (BOD**_**5**_**):** The biochemical oxygen demand over five days (BOD_5_) was determined by measuring the DO levels before and after a five-day incubation period using the DO meter. Initially, the DO level of the water sample was recorded. The sample was then incubated in a BOD bottle at 20 °C for five days in the dark to prevent photosynthesis. After the incubation period, the DO level was measured again. The BOD_5_ value was calculated by subtracting the final DO reading from the initial DO reading.

**Chemical Oxygen Demand (COD):** The chemical oxygen demand (COD) of water samples was determined using the dichromate reflux method. 2.5 mL of the water sample was placed in a reflux flask. Potassium dichromate (K_2_Cr_2_O_7_) solution (1.5 mL) and concentrated sulfuric acid (H_2_SO_4_) (3.5 mL) containing a silver sulfate catalyst were added to the sample. The mixture was then refluxed and heated for 2 h to ensure complete oxidation of the organic matter present in the sample. After the digestion process, the sample was allowed to cool to room temperature and then diluted with distilled water to a specific volume. The absorbance of the digested sample was measured using a DR-3900 spectrophotometer at a wavelength of 620 nm, which is specific to the dichromate ion. The absorbance values obtained were then used in the calibration curve equation: y = 2603.8x − 52.626, where x is the absorbance, to calculate the COD value. This equation was derived from a calibration curve with an R^2^ value of 0.9935, indicating a high level of accuracy.

**Nitrate (NO**_**3**_**⁻):** The concentration of nitrate (NO_3_⁻) in water samples was determined using the NitraVer 5 reagent, followed by spectrophotometric measurement. A 10 mL aliquot of the water sample was placed in a clean sample cell. The contents of one NitraVer 5 nitrate reagent powder pillow were added to the sample cell, and the cell was capped and shaken vigorously to ensure thorough mixing and complete reaction. The instrument timer was started, and a reaction time of 5 min was allowed for the sample. Simultaneously, a blank was prepared by filling a second sample cell with 10 mL of the water sample without adding any reagent. After the reaction time elapsed, the blank sample cell was cleaned and inserted into the spectrophotometer cell holder. The instrument was zeroed, displaying 0 mg/L NO_3_⁻. The prepared sample cell was then cleaned and inserted into the spectrophotometer within 5 min after the reaction time expired. The absorbance of the sample was measured using the spectrophotometer, and the nitrate concentration was displayed in mg/L NO_3_⁻.

**Sulfate (SO**_**4**_^**2**^**⁻):** The concentration of sulfate in water samples was determined using the SulfaVer 4 reagent, followed by spectrophotometric measurement. A 10 mL aliquot of the water sample was placed in a clean sample cell. The contents of one SulfaVer 4 sulfate reagent powder pillow were added to the sample cell, and the cell was capped and swirled to mix thoroughly. The instrument timer was started, and a reaction time of 5 min was allowed for the sample. Simultaneously, a blank was prepared by filling a second sample cell with 10 mL of the water sample without adding any reagent. After the reaction time elapsed, the blank sample cell was cleaned and inserted into the spectrophotometer cell holder. The instrument was zeroed, displaying 0 mg/L SO_4_^2^⁻. The prepared sample cell was then cleaned and inserted into the spectrophotometer within 5 min after the reaction time expired. The absorbance of the sample was measured using the spectrophotometer, and the sulfate concentration was displayed in mg/L SO_4_^2^⁻.

**Chloride (Cl⁻) and Sodium (Na⁺):** The concentration of chloride (Cl⁻) in water samples was determined using the Mohr titration method. A 50 mL aliquot of the water sample was placed into a clean conical flask. To this sample, 1.0 mL of potassium chromate indicator solution was added, resulting in a slight yellow coloration of the mixture. The sample was then titrated with a standard silver nitrate solution. The titration process involved the gradual addition of the silver nitrate solution to the sample while continuously swirling the flask. The endpoint of the titration, indicated by a brick-red color change, signified the complete precipitation of chloride ions as silver chloride (AgCl). The volume of silver nitrate solution used to reach this endpoint was carefully recorded. The concentration of chloride ions in the sample was calculated using Equation (3):

(3) $$\text{Chloride}\;\text{ion}\;\text{concentration}\;\left(\text{mg}/L\right)=\left(\frac{\;\text{Volume}\;\text{of}\;AgNO\;\text{used}\;\left(mL\right)-0.2}{\;\text{Volume}\;\text{of}\;\text{sample}\;\left(mL\right)}\right)\times 500$$

The concentration of sodium (Na⁺) in water samples was calculated based on the chloride (Cl⁻) concentration determined from the Mohr titration method. Sodium concentration was estimated using the formula: Na^⁺^ concentration = 0.648 × Cl^⁻^ concentration.

**Phosphate (PO**_**4**_^**3**^**⁻):** The concentration of phosphate in water samples was determined using the Cosber 3 reagent, followed by spectrophotometric measurement. A 10 mL aliquot of the water sample was placed in a clean sample cell. The contents of one Cosber 3 reagent powder pillow were added to the sample cell, and the cell was capped and shaken vigorously to ensure thorough mixing and complete reaction. The instrument timer was started, and a reaction time of 5 min was allowed for the sample. Simultaneously, a blank was prepared by filling a second sample cell with 10 mL of the water sample without adding any reagent. After the reaction time elapsed, the blank sample cell was cleaned and inserted into the spectrophotometer cell holder. The instrument was zeroed, displaying 0 mg/L phosphate. The prepared sample cell was then cleaned and inserted into the spectrophotometer within 5 min after the reaction time expired. The absorbance of the sample was measured using the spectrophotometer, and the phosphate concentration was displayed in mg/L phosphate.

**Arsenic:** The measurement of arsenic in water samples was conducted using a colorimetric analysis, where arsenic concentration was determined by the intensity of color developed on mercuric bromide paper. A 75 mL water sample was collected and prepared for analysis. The procedure involved adding sulfamic acid and zinc dust to the sample, facilitating the reduction of arsenic compounds into arsine gas. The sealed reaction vessel was left undisturbed for 20 min, allowing the arsine gas to react with the mercuric bromide paper, which changed color according to the arsenic concentration. The resulting color was then compared to a standardized chart with values ranging from 10 to 500 parts per billion (ppb). If no color change occurred, it indicated that the arsenic concentration was below the detection limit of 10 ppb, suggesting an extremely low or undetectable level of arsenic in the sample.

**Iron (Fe):** The measurement of iron (Fe) concentration in water samples was conducted using the MITK iron test kit. The procedure involved initiating Program 260 on the DR-3900 spectrophotometer and selecting the appropriate method. A 10 mL water sample was then placed into a clean 1-inch cell, which was subsequently inserted into the square cell holder. The instrument was calibrated to zero using the blank sample. Following this, 5 to 6 drops of Ferrozine solution were added to the water sample, which was then mixed thoroughly to ensure complete reaction. The reaction time was set, and after a 5-min period, the “Read” function on the spectrophotometer was activated to display the iron concentration in the sample.

**Manganese (Mn):** The measurement of manganese (Mn) concentration in water samples was performed using the MITK iron test kit and the DR-3900 spectrophotometer. The procedure began by initiating Program 295 on the DR-3900 and selecting the appropriate method for high-range manganese analysis. A 10 mL water sample was placed in a clean 1-inch cell, and one drop of Mn-BUF-1 buffer solution was added to the cell. The solution was mixed thoroughly to ensure proper reaction. The cell was then inserted into the square cell holder of the spectrophotometer, and the instrument was calibrated to zero using the blank sample. Subsequently, one drop of Mn-SMP-2 reagent was added to the sample, and the mixture was stirred. The reaction was allowed to proceed for 2 min, after which the “Read” function was activated to display the manganese concentration.

**Fecal Coliform (FC) and Total Coliform (TC):** To determine fecal coliform (FC) and total coliform (TC) counts, a 100 mL water sample was filtered through a sterilized Sartorius filter paper with a pore size of 0.45 μm for each analysis. The filter was then placed on the respective culture media: XMG Agar for FC and m-Endo Agar for TC. For FC analysis, Petri dishes containing XMG Agar were incubated at 44.5 °C for 24 h in a bacteriological incubator, where blue-green colonies were counted as fecal coliforms. For TC analysis, Petri dishes containing m-Endo Agar were incubated at 35 °C for 24 h, and typical coliform colonies, with or without a metallic sheen, were counted as total coliforms. Colony counts were expressed as colony-forming units (CFU) per 100 mL of water. To ensure accurate incubation conditions, the incubators were equipped with thermostats to maintain precise temperature control throughout the incubation period. The temperature was rigorously monitored, with checks conducted every hour for the first 8 h, and a final check performed just before the end of the incubation period. Efforts were made to avoid frequent opening of the incubator to prevent temperature fluctuations and ensure consistent incubation conditions.

#### 2.5.2 Comparison with standard guidelines

The parameters measured in the surface water samples, referred to as SW-1 and SW-2, were assessed against established standard values for the quality of irrigation water. These standards were obtained from reputable sources such as the Environmental Conservation Rule (ECR, 2023), Surface Water Quality Standards (IS: 2296), and relevant scientific literature. The samples of wastewater or mine drainage water (MDW-3 and MDW-4) were evaluated against established standard values for their disposal into the environment. These standards were obtained from the Environmental Conservation Rule (ECR, 2023), specifically the general standards for effluent discharge, and relevant research articles reviewed by experts in the field. The drinking water samples collected from hand tubewells (DW-5) and deep tubewells (DW-6) were compared against the standard values prescribed by IS: 10500 for the quality of drinking water.

#### 2.5.3 WQI analysis of drinking water

The Water Quality Index (WQI) is a numerical representation of overall water quality. It combines multiple water quality parameters that are relevant to specific purposes such as drinking, irrigation, or recreation, enabling comparison with established standards. The concept of WQIs was introduced by Horton, who proposed an eight-parameter index for water system assessment [29]. The most commonly used method for calculating WQI employs the formula shown in Equation (4) [30]:

(4) $$\text{WQI}=\sum_{i=1}^{n}\;Q_{i}W_{i}/\sum_{i=1}^{n}\;W_{i}$$

where.

➢ *Q*_*i*_ = sub-index for the *i-*th parameter (representing its quality rating)
➢ *W*_*i*_ = weight assigned to the ith parameter
➢ *n* = total number of parameters considered

Calculation of *Q*_*i*_ value:

The *Q*_*i*_ value, which represents the quality rating for the *i*-th parameter, is calculated using the formula shown in Equation (5):

(5) $$Q_{i}=\left[\left(V_{i}-V_{o}\right)/\left(S_{i}-V_{o}\right)\right]$$

where:

*V*_*i*_ = measured value of the ith parameter.

*S*_*i*_ = standard permissible value for the ith parameter.

*V*_*0*_ = ideal value of the ith parameter in pure water (*V*_*0*_ = 7.0 for pH, *V*_*0*_ = 14.6 mg/L for dissolved oxygen, *V*_*0*_ = 0 for all other parameters) [31].

Calculation of *W*_*i*_ value:

The weight assigned to each parameter (*W*_*i*_) is inversely proportional to the recommended standard for that parameter.

$$W_{i}\propto 1/S_{i}\;or\;W_{i}=K/S_{i}$$

Where,

$$K=\frac{1}{\sum_{i=1}^{n}\;\frac{1}{Si}}$$

The WQI typically ranges from 0 to 100, with lower values indicating better water quality. Classification schemes categorize WQI values into different water quality categories (e.g., excellent, good, poor, very poor, unfit) based on pre-defined ranges (Table 2).

**Table 2:** Classification of water quality based on the WQI.

Water quality index	Water quality status	Grading
0–25	Excellent	A
26–50	Good	B
51–75	Poor	C
76–100	Very poor	D
>100	Unfit for consumption	E

### 2.6 Collection of soil samples

Soil samples were obtained from three distinct locations near the Barapukuria coal mining area during both the summer (May 2023) and winter (December 2023) seasons. The samples were collected by scraping them vertically from the top to the bottom using an auger at a depth of 15–20 cm. For each sample, 500 g of soil was taken. Each sample was collected in the afternoon and clearly labeled with the specific date, location, and corresponding identifier: NDS-7 for surface soil near mine drainage, CTS-8 for agricultural land farmed with surface water near the mine drainage, and NFS-9 for normal field soil farmed with groundwater. From the collected samples, any gravels, pebbles, plant roots, leaves, etc. were removed and preserved in polythene bags for laboratory analysis.

### 2.7 Soil sample analysis

#### 2.7.1 Analytical methods

The soil samples were transported under ambient conditions and air-dried at room temperature for seven days upon arrival. This approach is in accordance with standard soil sample preparation practices, which involve stabilizing the moisture content to ensure the suitability of the samples for analysis. Subsequently, the dried samples were crushed using a mechanical grinder. The crushed soil was then passed through a 2-mm stainless steel sieve to achieve a uniform particle size for further analysis. Careful transportation was employed to transfer the sieved soil samples to the SRDI for a comprehensive analysis of various physicochemical properties. These properties encompassed soil pH, total organic matter (OM), available phosphorus (P), available micronutrients (Zn, Fe, Mn, B, Cu, Pb, Cd), exchangeable cations (K, Ca, Mg), and available sulfur (S). The specific methods utilized by SRDI for each analysis are shown in Table 3. Upon receipt of the analytical results from SRDI, the data were compiled, tabulated, and analyzed using Microsoft Office Excel software.

**Table 3:** Methods used for soil property analysis.

Soil property	Measurement method
Soil pH	Glass electrode method (soil: water ratio 1:2.5)
Organic matter	Walkley and Black's winter oxidation method
Available phosphorus	Olsen method
Available zinc	DTPA method
Available iron	DTPA micronutrient extraction method
Available copper	DTPA micronutrient extraction method
Available manganese	DTPA micronutrient extraction method
Exchangeable potassium	Ammonium acetate extraction method
Available lead	1:5 nitric acid extraction method
Available cadmium	1:5 nitric acid extraction method
Exchangeable calcium	EDTA titration method
Exchangeable magnesium	EDTA titration method

#### 2.7.2 Geo-accumulation index (I_geo_) of surface soil

The I_geo_ introduced by Muller (1969), is a widely used method for assessing metal contamination in sediments and soils. It is calculated using Equation (6):

(6) $$I_{geo\;}=\log_{2}\;\left[\frac{C_{metal\;\left(sample\right)\;}}{1.5\times C_{metal\;\left(background\right)\;}}\right]$$

where.

➢ *C*_*metal*_ = Concentration of the analyzed metal in the sample (μg/g)
➢ *C*_*metal (background)*_ = Geochemical background value for the specific metal (μg/g)
➢1.5 = Background matrix correction factor to account for natural variations in background levels [32].

Halim et al. [33] reported background metal concentrations (μg/g) in uncontaminated soils collected near the Barapukuria mining area (approximately 5 km distance). These values were: Cu (8.37), Pb (22.47), Cd (0.17), Zn (52.74), and Fe (20,696). Table 4 summarizes the seven contamination classes defined by the I_geo_, with an index ranging from 0 to 6 [34]. Each class corresponds to a specific I_geo_ value range and its associated level of soil contamination.

**Table 4:** The seven classes of the geo-accumulation index.

Index class	I_geo_ Value	Level of contamination classification
0	I_geo_ < 0	Uncontaminated
1	0 ≤ I_geo_ < 1	Uncontaminated to moderately contaminated
2	1 ≤ I_geo_ < 2	Moderately contaminated
3	2 ≤ I_geo_ < 3	Moderately to heavily (strongly) contaminated
4	3 ≤ I_geo_ < 4	Heavily (strongly) contaminated
5	4 ≤ I_geo_ < 5	Heavily (strongly) to extremely contaminated
6	I_geo_ ≥ 5	Extremely contaminated

#### 2.7.3 Soil fertility analysis

Coal water treated soil (CTS-8) was assessed in accordance with the guidelines set by the Bangladesh Agricultural Research Council (BARC) to evaluate its soil fertility. The analysis encompassed determining pH levels, measuring total organic matter, and assessing nutrient contents.

## 3 Results and discussion

### 3.1 Quality evaluation of water

#### 3.1.1 Surface water

##### 3.1.1.1 Suitability for irrigation

**Fig. 3:**
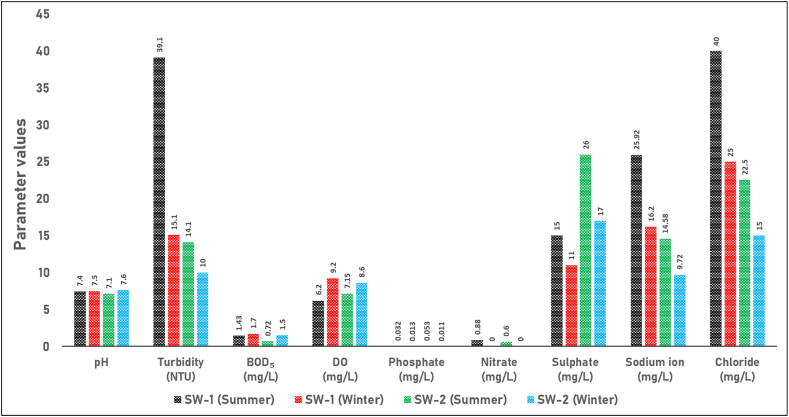
Seasonal variations in water quality parameters of surface water samples (SW-1 and SW-2). The figure illustrates the levels of pH, Turbidity (NTU), Biochemical Oxygen Demand (BOD_5_) (mg/L), Dissolved Oxygen (DO) (mg/L), Phosphate (mg/L), Nitrate (mg/L), Sulfate (mg/L), Sodium ion (mg/L), and Chloride (mg/L) in surface water samples SW-1 and SW-2 during the summer and winter seasons.

Fig. 3 illustrates the seasonal variations in pH, turbidity, biochemical oxygen demand, dissolved oxygen, phosphate, nitrate, sulfate, sodium ions, and chloride in surface water samples (SW-1 and SW-2) collected during the summer and winter seasons. The pH values of both surface water samples in both seasons fall within the acceptable range of 6.5–8.5, which is considered suitable for irrigation purposes. According to research on water quality for irrigation, turbidity levels exceeding 5 NTU can have a negative impact on crop growth [35]. The analysis of turbidity levels in the surface water samples (SW-1 and SW-2) reveals that both sites exceeded the 5 NTU threshold in both summer and winter. During the summer, turbidity in SW-1 reached 39.10 NTU, while SW-2 recorded 14.10 NTU. Even in winter, turbidity levels remained above the threshold, with SW-1 at 15.10 NTU and SW-2 at 10.00 NTU. High turbidity levels reduce light penetration, which is crucial for photosynthesis, and can lead to soil pore clogging, thereby impeding water absorption by plants [36]. Research indicates that optimal dissolved oxygen (DO) levels for irrigation range between 5 and 8 mg/L, as these levels support aerobic microbial activity crucial for nutrient cycling and plant growth [37]. The DO levels in both samples during the summer and winter seasons fell within this optimal range, suggesting a conducive environment for microbial activity and overall plant health. Furthermore, the phosphate concentrations of both surface water samples in both seasons are well below the standard value of 2 mg/L, indicating that the water is suitable for irrigation in terms of phosphate content. On the other hand, the nitrate concentrations of both samples in both seasons are significantly lower than the standard value of 5 mg/L, suggesting that the water is suitable for irrigation purposes in terms of nitrate content. Additionally, the sulfate concentrations of both surface water samples in both seasons are much lower than the standard value of 1000 mg/L, indicating that the water is suitable for irrigation in terms of sulfate content. The sodium concentrations in the surface water samples from both seasons were well below the standard value of 200 mg/L, suggesting that the water is suitable for irrigation purposes in terms of sodium content. Similarly, the chloride levels in the surface water samples from both seasons were significantly lower than the standard value of 600 mg/L, indicating that the water is suitable for irrigation in terms of chloride content.

**Fig. 4:**
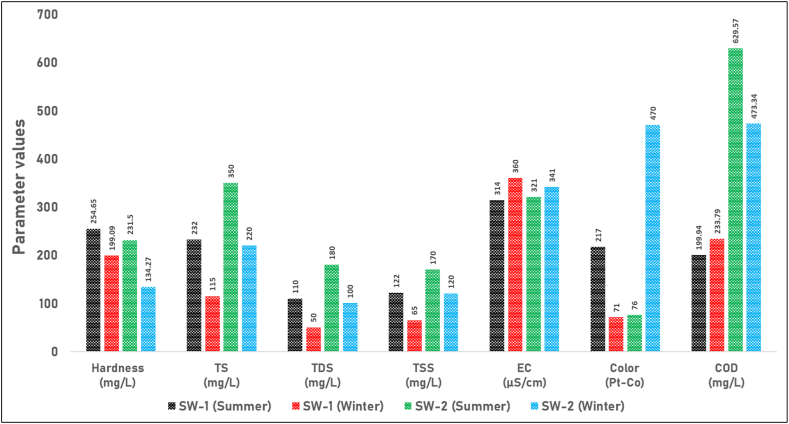
Seasonal variations in water quality parameters of surface water samples (SW-1 and SW-2). The figure illustrates the levels of Hardness (mg/L), Total Solids (TS) (mg/L), Total Dissolved Solids (TDS) (mg/L), Total Suspended Solids (TSS) (mg/L), Electrical Conductivity (EC) (μS/cm), Color (Pt-Co), and Chemical Oxygen Demand (COD) (mg/L) in surface water samples SW-1 and SW-2 during the summer and winter seasons.

Fig. 4 illustrates the variations in hardness, TS, TDS, TSS, EC, color, and COD in surface water samples (SW-1 and SW-2) collected during the summer and winter seasons. For SW-1, the hardness value decreases from 254.65 mg/L in the summer season to 199.09 mg/L in the winter season. Similarly, SW-2 shows a decrease in hardness from 231.50 mg/L during the summer season to 134.27 mg/L in the winter season. High hardness levels in irrigation water can have both beneficial and detrimental effects on agricultural practices. The elevated concentrations of calcium and magnesium, which contribute to water hardness, can improve soil structure by enhancing soil aggregation and increasing porosity [38]. This improved soil structure can facilitate root growth and enhance nutrient availability, potentially benefiting crop growth. However, excessive hardness can lead to issues such as nutrient imbalances, where high levels of calcium and magnesium interfere with the uptake of other essential nutrients like potassium and phosphorus. Additionally, high hardness can reduce water permeability in soils, leading to poor water infiltration and drainage, which can negatively impact plant health and yield [39]. Although there is no standard value provided for TS in irrigation water, elevated levels can negatively impact soil permeability and plant growth [40]. High TS levels mean that the water contains a significant amount of dissolved and suspended solids, such as silt, clay, organic matter, and salts. When this water is used for irrigation, these particles can clog soil pores, reducing the soil's ability to absorb and drain water. This can lead to waterlogging, where the soil becomes oversaturated and lacks proper aeration, affecting root health and reducing the effectiveness of irrigation. The TDS values of both surface water samples in both seasons were well below the standard value of 1000 mg/L, indicating that the water is suitable for irrigation in terms of TDS content. Although there is no specific standard for TSS in irrigation water, excessive levels can clog emitters, nozzles, and sprinklers, reducing the efficiency of irrigation systems and leading to uneven water distribution [41]. This uneven distribution can result in over-irrigation or under-irrigation, causing waterlogging or drought stress in plants, both of which negatively impact plant health and yield. Additionally, high TSS levels can lead to soil compaction and reduced permeability, hindering root growth and water infiltration, and potentially causing nutrient imbalances that further impair plant development [42]. The EC values of both surface water samples in both seasons were significantly lower than the standard value of 2250 μS/cm, suggesting that the water is suitable for irrigation in terms of electrical conductivity. While there is no standard value provided for color in irrigation water, high levels can indicate the presence of organic matter, dissolved solids, or other contaminants that may affect plant growth and soil quality [43]. The COD values of both surface water samples in both seasons exceeded the standard value of 100 mg/L, indicating the presence of organic matter and other oxidizable substances. Elevated COD levels in irrigation water can negatively impact soil and plant health by reducing oxygen availability in the soil, leading to poor root respiration and potential root diseases. Additionally, high COD can introduce harmful compounds that may be toxic to plants, disrupt nutrient uptake, and alter soil microbial communities, further impairing plant growth and soil quality [44].

##### 3.1.1.2 Seasonal variations

The analysis of pH values revealed a slight increase from summer to winter for both surface water samples. However, this difference was minimal, and the values remained within the prescribed standard range during both seasons. In contrast, the turbidity values showed noticeable seasonal variations. Higher turbidity levels were observed in the summer compared to the winter for both surface water samples. This variation could be attributed to factors such as increased surface runoff, elevated suspended sediment concentrations, or enhanced algal growth during the warmer summer season [45]. These factors contribute to the higher turbidity by increasing the amount of particulate matter and organic material in the water. Additionally, the biochemical oxygen demand (BOD_5_) values showed a slight increase from summer to winter for both surface water samples. This indicates a marginal increase in organic loading during the winter period. Furthermore, the dissolved oxygen (DO) concentrations exhibited an increasing trend from summer to winter for both surface water samples. This could be related to factors such as lower water temperatures and increased dissolved oxygen solubility during the colder winter season [46]. As temperatures drop, the solubility of oxygen in water increases, allowing more oxygen to be dissolved in the water. Additionally, reduced biological activity in colder months means less oxygen is consumed by organisms, contributing to higher DO levels. The parameters related to phosphate, nitrate, sulfate, sodium ion, and chloride generally exhibit a decreasing trend from summer to winter in both surface water samples. However, the degree of seasonal variation varies among these parameters. It is worth noting that the hardness values of both surface water samples also show a decreasing trend from summer to winter. This can be attributed to factors such as dilution effects from precipitation or changes in water sources [47]. During the winter season, increased rainfall can lead to higher water volumes, which dilute the concentration of hardness-causing minerals like calcium and magnesium. Additionally, seasonal shifts in water sources, such as increased inflow from softer bodies of water or groundwater, can contribute to the observed decrease in hardness. Similarly, the values of both total solids (TS) and total dissolved solids (TDS) exhibit a decreasing trend from summer to winter in both surface water samples. This may be due to dilution effects or lower evaporation rates during the cooler winter season [48]. In winter, increased precipitation and runoff can result in a greater volume of water, which dilutes the concentration of solids. Furthermore, cooler temperatures lead to reduced evaporation rates, meaning less water is lost to the atmosphere and, consequently, the concentration of dissolved and suspended solids remains lower. The total suspended solids (TSS) values also decreased from summer to winter for both surface water samples, which may be attributed to reduced surface runoff and sediment loading during the winter period [49]. In the summer, heavy rainfall and increased surface runoff can transport more sediments and organic matter into water bodies, elevating TSS levels. Conversely, in winter, decreased rainfall and runoff result in fewer sediments being washed into the water, leading to lower TSS concentrations. On the other hand, the electrical conductivity (EC) values slightly increased from summer to winter for both surface water samples. This may be attributed to changes in the concentration of dissolved ionic species or other factors influencing conductivity [50]. As water temperatures decrease in winter, the solubility of certain salts and minerals can increase, causing higher concentrations of dissolved ions that enhance electrical conductivity. Moreover, decreased biological activity and reduced nutrient uptake by organisms during the colder months may leave more ions dissolved in the water. Regarding color, divergent trends were observed between the two surface water samples. The color of the SW-1 sample decreased from summer to winter, whereas the color of the SW-2 sample significantly increased during the winter season. This variation could be influenced by factors such as changes in organic matter inputs, the occurrence of algal blooms, or other seasonal processes affecting the water bodies [51]. For the SW-1 sample, the decrease in color may be attributed to reduced input of organic matter and lower biological activity during the colder months. On the other hand, the increase in color of the SW-2 sample could be due to the accumulation of decaying organic material or the presence of specific types of algae that thrive in cooler temperatures. Seasonal runoff patterns, variations in water flow, and different watershed characteristics may also play a role in these observed trends. Additionally, the chemical oxygen demand (COD) values indicate a decreasing trend for the SW-2 water sample and an increasing trend from summer to winter for SW-1. This suggests an increase in organic matter and oxidizable substances during the winter season for SW-1 [52]. The decrease in COD values for SW-2 suggests a reduction in organic pollutants or more efficient natural degradation processes during the winter months. This could be attributed to factors such as less organic runoff, decreased biological activity, or improved dilution effects. Conversely, the increase in COD values for SW-1 implies a higher concentration of organic matter and other oxidizable substances in this water sample during the colder season. This increase could be due to the accumulation of decaying vegetation, reduced microbial degradation rates, or other seasonal factors that contribute to higher levels of organic pollutants.

#### 3.1.2 Wastewater

##### 3.1.2.1 Suitability for disposal

**Fig. 5:**
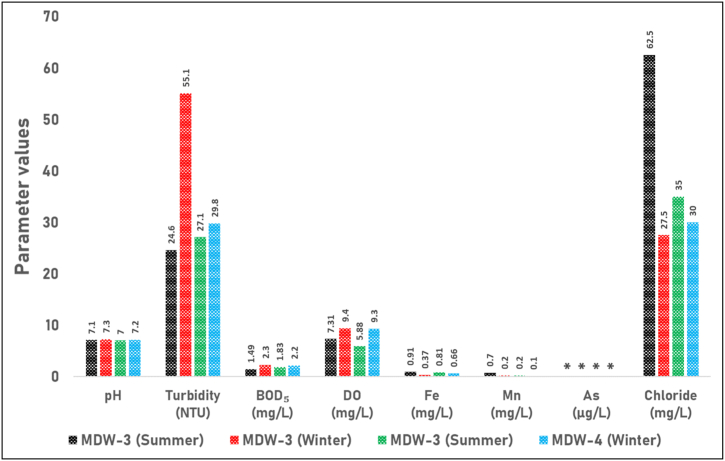
Seasonal variations in water quality parameters of wastewater samples (MDW-3 and MDW-4). The figure illustrates the pH, Turbidity (NTU), Biochemical Oxygen Demand (BOD_5_) (mg/L), Dissolved Oxygen (DO) (mg/L), Iron (Fe) (mg/L), Manganese (Mn) (mg/L), Arsenic (As) (μg/L), and Chloride (mg/L) concentrations in wastewater samples MDW-3 and MDW-4 during the summer and winter seasons. ‘**∗**’ indicates Below Detection Limit, meaning that the arsenic concentration was too low to be detected by the method used.

Fig. 5 shows the seasonal variations in pH, turbidity, BOD_5_, DO, Fe, Mn, As, and chloride concentrations in the wastewater samples (MDW-3 and MDW-4) during the summer and winter seasons. The pH values of both samples fall within the acceptable range of 6–9, which indicates that the wastewater is suitable for disposal based on this parameter. However, the turbidity values exceeded the standard of 10 NTU in both seasons for both MDW-3 and MDW-4, indicating the need for treatment to reduce turbidity levels before disposal. The DO concentrations of MDW-3 in both seasons and MDW-4 in the winter season were within the standard range of 4.5–8 mg/L, indicating appropriate oxygen levels. However, the DO value of MDW-4 in the summer season (5.88 mg/L) slightly exceeded the standard range. The BOD_5_ values of both samples were well below the standard of 30 mg/L in both seasons, indicating a low organic load and suitability for disposal in terms of BOD_5_. In addition, the Fe concentrations in both MDW-3 and MDW-4 samples were below the standard of 3 mg/L in both seasons, indicating that the wastewater is suitable for disposal in terms of iron content. Similarly, the Mn concentrations in both samples were below the standard of 2 mg/L in both seasons, suggesting the suitability of the wastewater in terms of manganese content. The As concentrations in both MDW-3 and MDW-4 samples were within the standard of 0.2 mg/L in both seasons, indicating the suitability of the wastewater for disposal in terms of arsenic content. Furthermore, the Cl⁻ concentrations in both samples were well below the standard of 600 mg/L in both seasons, suggesting the suitability of the wastewater for disposal in terms of chloride content. However, the color values of both MDW-3 and MDW-4 samples were significantly higher than the typical range for drinking water, indicating the need for treatment to reduce color before disposal.

**Fig. 6:**
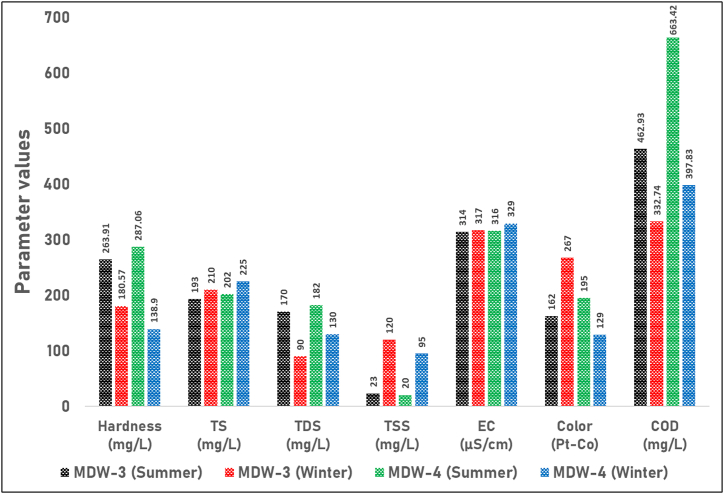
Seasonal variations in water quality parameters of wastewater samples (MDW-3 and MDW-4). The figure illustrates the levels of Hardness (mg/L), Total Solids (TS) (mg/L), Total Dissolved Solids (TDS) (mg/L), Total Suspended Solids (TSS) (mg/L), Electrical Conductivity (EC) (μS/cm), Color (Pt-Co), and Chemical Oxygen Demand (COD) (mg/L) in wastewater samples MDW-3 and MDW-4 during the summer and winter seasons.

Similarly, Fig. 6 shows the seasonal variations in hardness, TS, TDS, TSS, EC, color, and COD values in the wastewater samples (MDW-3 and MDW-4) during the summer and winter seasons. Both samples exhibited hardness values below the standard threshold of 500 mg/L in both seasons, indicating that the wastewater was suitable for disposal in terms of hardness. Moreover, the TS values of both MDW-3 and MDW-4 were well below the standard limit of 2100 mg/L in both seasons, implying that the wastewater was suitable for disposal in terms of total solids content. Notably, the TDS values of both samples were significantly lower than the standard limit of 2100 mg/L in both seasons, demonstrating that the wastewater was suitable for disposal in terms of dissolved solids content. In the case of the wastewater sample MDW-3, the TSS value of 23 mg/L during the summer season adhered to the standard limit of 100 mg/L, indicating good water quality during this period. However, during the winter season, the TSS value of 120 mg/L exceeded the standard limit, suggesting compromised water quality due to elevated levels of suspended solids. Conversely, for MDW-4, the summer TSS value of 20 mg/L was well below the standard limit, indicating good water quality. However, the winter TSS value of 95 mg/L was slightly below the limit but relatively higher compared to the summer season. Furthermore, both MDW-3 and MDW-4 samples exhibited COD values that exceeded the standard threshold of 200 mg/L in both seasons, indicating a high organic load in the wastewater that may require treatment prior to disposal. Although no specific standard value was provided for EC, the values of both MDW-3 and MDW-4 samples fell within the typical range for freshwater, suggesting that the wastewater was suitable for disposal in terms of conductivity.

##### 3.1.2.2 Seasonal variations

The pH values of both wastewater samples, MDW-3 and MDW-4, exhibited a slight increase from summer to winter. However, these variations were minimal and remained within an acceptable range. In contrast, the turbidity values for both samples demonstrated a significant increase from summer to winter, indicating higher levels of suspended solids or particulate matter during the winter season. The dissolved oxygen (DO) concentrations in both MDW-3 and MDW-4 samples also increased from summer to winter. This can be attributed to the lower water temperatures and higher oxygen solubility during the colder months. As temperatures decrease, the solubility of oxygen in water naturally increases, allowing for more oxygen to be dissolved. Moreover, cooler temperatures often result in decreased metabolic rates in aquatic organisms, leading to lower consumption of dissolved oxygen. Reduced biological activity and respiration rates during the winter further contribute to higher DO levels. Additionally, the biochemical oxygen demand (BOD_5_) values slightly increased from summer to winter for both samples, suggesting a slight increase in organic loading during the winter season. Conversely, the iron (Fe) concentrations showed a decreasing trend from summer to winter for both MDW-3 and MDW-4 samples. This may be due to changes in redox conditions or precipitation or adsorption processes [53]. During the winter season, lower temperatures can impact the redox conditions, leading to the oxidation of dissolved iron into particulate forms that precipitate out of the water column. Furthermore, the reduced biological activity and slower metabolic rates during colder months can result in a decrease in the release of iron from sediments. At lower temperatures, adsorption processes may also become more efficient as iron ions bind more readily to particulate matter and settle out of the water. Similarly, the concentrations of manganese (Mn) exhibited a significant decrease from summer to winter in both samples, possibly as a result of changes in redox conditions or precipitation/adsorption processes [53]. No seasonal variation was observed in arsenic (As) concentrations, as the values remained consistently zero in both seasons for both MDW-3 and MDW-4 samples. The concentrations of chloride (Cl⁻) showed a noticeable decrease from summer to winter in MDW-3, while a slight decrease was observed in MDW-4. This could be attributed to dilution effects or changes in groundwater flow patterns [54]. Increased winter precipitation can result in higher water volumes, leading to a dilution of chloride concentrations in surface and groundwater sources. Moreover, alterations in groundwater flow patterns during colder months may decrease the entry of chloride-rich waters into the sampling areas. Seasonal variations in land use, runoff, and reduced evaporation rates can also contribute to the observed declines in chloride levels. Furthermore, there were contrasting trends in color values, with an increase from summer to winter for MDW-3, while MDW-4 showed a decrease. This suggests potential seasonal variations in the sources or processes influencing the color of wastewater. The hardness values exhibited a decline from summer to winter for both MDW-3 and MDW-4 samples, which could be linked to changes in water chemistry, mineral dissolution, or precipitation processes. Total solids (TS) values showed a slight increase from summer to winter for both MDW-3 and MDW-4 samples, indicating a higher concentration of solids during the winter period. In contrast, total dissolved solids (TDS) values demonstrated a significant decrease from summer to winter for both samples, potentially due to dilution effects or changes in groundwater flow patterns [55]. During the winter months, increased precipitation and reduced evaporation rates can result in higher water volumes, leading to a dilution of dissolved solids in surface and groundwater sources. Moreover, seasonal changes in groundwater flow patterns can affect the influx of mineral-rich waters, contributing to the observed decrease in Total Dissolved Solids (TDS) levels. The values of Total Suspended Solids (TSS) demonstrated a noteworthy increase from summer to winter in both MDW-3 and MDW-4 samples, indicating elevated levels of suspended solids or particulate matter during the winter period. This increase may be linked to heightened surface runoff or erosion [56]. In winter, the combination of increased precipitation and reduced vegetation can lead to higher rates of surface runoff, which carries more sediment and particulate matter into water bodies. Additionally, winter storms and melting snow can contribute to erosion, further increasing TSS levels. The values of Chemical Oxygen Demand (COD) displayed a decreasing trend from summer to winter in both MDW-3 and MDW-4 samples, suggesting a lower organic load in the wastewater during the winter period. Finally, the values of electrical conductivity (EC) exhibited a slight increase from summer to winter in both the MDW-3 and MDW-4 samples, which could be attributed to changes in water chemistry or dissolved solids content.

#### 3.1.3 Drinking water

##### 3.1.3.1 Overall quality and seasonal variations

Table 5 presents the values of various parameters measured in samples collected from hand tubewell (DW-5) and deep tubewell (DW-6) during both the summer and winter seasons. The pH levels were found to be within the acceptable range of 6.5–8.5 for both sources and seasons. However, the turbidity exceeded the permissible limit of 5 NTU in the hand tubewell during the summer, indicating higher levels of particulate matter during this period. While the hardness and total dissolved solids (TDS) values were within the prescribed limits, the presence of coliform bacteria, particularly in the hand tubewell, made the water unsafe for drinking without treatment. The total coliform (TC) and fecal coliform (FC) levels in the hand tubewell (DW-5) and deep tubewell (DW-6) samples demonstrate minor seasonal variations. In the hand tubewell (DW-5), TC levels decrease from 25 N/100 mL in the summer to 12 N/100 mL in the winter, while FC levels drop from 8 N/100 mL to 3 N/100 mL over the same period. In the deep tubewell (DW-6), TC levels decline from 10 N/100 mL in the summer to 4 N/100 mL in the winter, with FC levels remaining consistently at 0 N/100 mL across both seasons. These findings are crucial for assessing the microbiological quality of drinking water. The presence of coliform bacteria, particularly fecal coliforms, indicates potential contamination by pathogens that can pose significant health risks [57]. The Indian Standard (IS) 10500 for drinking water specifies a strict standard of zero tolerance for both total coliforms and fecal coliforms. While the deep tubewell (DW-6) meets this standard for fecal coliforms, both water sources exceed the permissible limits for total coliforms in both seasons. The hand tubewell (DW-5) also fails to meet the standard for fecal coliforms in summer, although it approaches compliance in winter. Both tubewells showed elevated levels of iron and manganese, exceeding the standard values, with higher concentrations during the summer season. The chloride levels were acceptable, and there was no concern regarding arsenic contamination as the values consistently remained within the permissible limit of 10 μg/L. Notably, there were seasonal variations, with parameters such as turbidity, hardness, TDS, coliforms, iron, and manganese showing higher values during the summer compared to the winter season.

**Table 5:** Water quality parameter values in drinking water.

Parameters	Season	Hand tubewell (DW-5)	Deep tubewell (DW-6)	Standard value (IS: 10500)
pH	Summer	6.90	7.10	6.5–8.5
	Winter	7.20	7.40	
Turbidity (NTU)	Summer	12.30	4.74	5
	Winter	3.26	3.26	
Hardness (mg/L)	Summer	189.83	97.23	300
	Winter	106.49	91.20	
TDS (mg/L)	Summer	180.00	104.00	500
	Winter	130.00	70.00	
TC (N/100 mL)	Summer	25	10	0
	Winter	12	4	
FC (N/100 mL)	Summer	8	0	0
	Winter	3	0	
Fe (mg/L)	Summer	1.64	0.31	0.3
	Winter	0.73	0.19	
Mn (mg/L)	Summer	1.30	0.40	0.1
	Winter	0.60	0.30	
Cl^−^ (mg/L)	Summer	77.50	45.00	250
	Winter	65.00	15.00	
As (μg/L)	Summer	BDL^a^	BDL^a^	10
	Winter	BDL^a^	BDL^a^	

a BDL: Below Detection Limit.

##### 3.1.3.2 WQI analysis

Table 6 presents the Water Quality Index (WQI) values and corresponding assessments for two sampling locations, DW-5 and DW-6, during the summer and winter. The evaluation of the Water Quality Index (WQI) for drinking water samples collected from a hand tubewell (DW-5) and a deep tubewell (DW-6) in the vicinity of the Barapukuria coal mining area provides valuable insights into the suitability of the water for consumption. For DW-5, the WQI value of 441.84 during the summer season indicates that the water is classified as unsuitable for consumption due to significant contamination and poor quality, necessitating treatment before any potential use. In the winter season, the WQI for DW-5 is 202.79, which also falls under the unsuitable category, indicating persistent contamination issues that require remediation, although the value is lower than in the summer. Regarding DW-6, the WQI in the summer season is 129.66, also categorized as unsuitable for consumption, highlighting the presence of contaminants at levels that pose potential health risks and the need for substantial treatment. In the winter season, the WQI for DW-6 decreases to 95.88, classified as very poor, representing an improvement compared to the summer season but still indicating inadequate water quality for safe consumption without proper treatment measures. The analysis of the WQI reveals that the drinking water quality in the vicinity of the Barapukuria coal mining area is generally unsuitable for consumption, with the hand tubewell (DW-5) experiencing significantly greater compromises in water quality compared to the deep tubewell (DW-6). Seasonal variations demonstrate higher levels of contamination during the summer compared to the winter. To ensure a safe drinking water supply, it is imperative to implement comprehensive water treatment processes and continuous monitoring to mitigate the adverse impacts of mining activities on water quality in the region.

**Table 6:** Results of water quality index (WQI).

Location	Season	WQI	Comment
DW-5	Summer	441.84	Unfit for consumption
	Winter	202.79	Unfit for consumption
DW-6	Summer	129.66	Unfit for consumption
	Winter	95.88	Very poor

### 3.2 Quality evaluation of soil

#### 3.2.1 Geo-accumulation index (I_geo_) analysis of surface soil

Table 7 presents the geo-accumulation index (I_geo_) values and corresponding classes for various heavy metals (Cu, Zn, Pb, Cd, and Fe) in surface soil samples collected near mine drainage (NDS-7) during the summer and winter seasons. The I_geo_ values provide a quantitative assessment of the level of heavy metal contamination in the soil. During the summer season, the I_geo_ values for Cu (−1.77), Zn (−3.12), Pb (−0.51), and Fe (−8.68) fall within class 0, indicating uncontaminated conditions for these heavy metals. However, the I_geo_ value for Cd (0.03) falls within class 1, suggesting uncontaminated to moderately contaminated conditions for cadmium during the summer season. In the winter season, the I_geo_ values for Cu (−2.49), Zn (−3.57), Pb (−0.77), and Fe (−9.49) remain in class 0, indicating uncontaminated conditions for these heavy metals. Notably, the I_geo_ value for Cd (−1.42) shifts to class 0, suggesting uncontaminated conditions for cadmium during the winter season. The seasonal variation in I_geo_ values for cadmium is noteworthy, with a higher value (class 1) observed during the summer season compared to the winter season (class 0). This variation could be attributed to factors such as increased surface runoff, changes in soil pH, or other environmental conditions during the summer season that may influence the mobility and bioavailability of cadmium in the soil. Overall, the data suggest that the surface soil near mine drainage (NDS-7) is generally uncontaminated by the analyzed heavy metals, with the exception of cadmium during the summer season, which exhibited uncontaminated to moderately contaminated conditions.

**Table 7:** Geo-accumulation index of surface soil near mine drainage.

Parameter	Season	NDS-7	I_geo_ value	I_geo_ class
Cu (μg/g)	Summer	3.67	−1.77	0
	Winter	2.23	−2.49	0
Zn (μg/g)	Summer	9.09	−3.12	0
	Winter	6.68	−3.57	0
Pb (μg/g)	Summer	23.63	−0.51	0
	Winter	19.74	−0.77	0
Cd (μg/g)	Summer	0.261	0.03	1
	Winter	0.095	−1.42	0
Fe (μg/g)	Summer	75.70	−8.68	0
	Winter	43.03	−9.49	0

#### 3.2.2 Comparison of coal water treated soil with normal field soil

Table 8 presents the soil quality parameters for coal water treated soil (CTS-8) and normal field soil (NFS-9) collected during the summer and winter seasons. The pH levels in CTS-8 exhibited an increase from summer (6.30) to winter (7.10), indicating a shift towards neutrality. NFS-9 also showed a similar trend, but with lower values of 5.90 in summer and 6.15 in winter. The organic matter (OM) content in CTS-8 decreased slightly from 7.91 % in summer to 7.17 % in winter, whereas NFS-9 exhibited consistently low OM levels of 1.62 % and 1.60 % in summer and winter, respectively. This suggests that soil irrigated with water near mine drainage significantly enhances soil organic matter content compared to normal field soil. The analysis revealed varying trends in the concentrations of essential micronutrients and heavy metals across the two soil types and seasons. The zinc (Zn) concentration in CTS-8 increased from summer (7.25 μg/g) to winter (7.81 μg/g), while NFS-9 showed a minor increase from 1.04 μg/g to 1.10 μg/g. Copper (Cu) levels in CTS-8 decreased from 4.40 μg/g in summer to 2.19 μg/g in winter, exhibiting a more significant seasonal fluctuation compared to NFS-9, where Cu levels were consistently lower, with a slight increase from 1.98 μg/g in summer to 1.25 μg/g in winter. Furthermore, the potassium (K) content in CTS-8 increased from 0.26 meq/100g in summer to 0.64 meq/100g in winter, suggesting that coal water treatment may enhance potassium availability, particularly in winter. In contrast, NFS-9 showed negligible change in K levels between seasons. The calcium (Ca) levels in CTS-8 were higher in summer (10.30 meq/100g) compared to winter (7.16 meq/100g), indicating seasonal depletion. NFS-9 exhibited lower Ca levels overall, with a similar seasonal trend but at lower concentrations. The phosphorus (P) concentration in CTS-8 increased from 9.76 μg/g in summer to 11.23 μg/g in winter, and NFS-9 also showed an increase, but at lower levels compared to CTS-8, indicating enhanced phosphorus retention in treated soil. Magnesium (Mg) levels in CTS-8 decreased from 2.13 meq/100g in summer to 1.27 meq/100g in winter, with a more pronounced decrease compared to NFS-9. The manganese (Mn) and iron (Fe) contents in CTS-8 exhibited higher concentrations and more significant seasonal variations compared to NFS-9, decreasing from summer to winter in both soil types. The sulfur (S) content in CTS-8 remained relatively stable, with 202.49 μg/g in summer and 209.79 μg/g in winter, while NFS-9 showed a slight increase from 25.33 μg/g in summer to 26.14 μg/g in winter. The consistently higher S levels in treated soil indicate an enrichment effect from coal water treatment. Overall, the analysis suggests that soil farmed with water near mine drainage significantly affects the soil quality parameters, leading to higher levels of organic matter, micronutrients, and pH in CTS-8 compared to NFS-9.

**Table 8:** Soil quality parameters of coal water treated soil and normal field soil.

Parameter	Unit	Season	CTS-8	NFS-9
pH	–	Summer	6.30	5.90
		Winter	7.10	6.15
OM	%	Summer	7.91	1.62
		Winter	7.17	1.60
Zn	μg/g	Summer	7.25	1.04
		Winter	7.81	1.10
Cu	μg/g	Summer	4.40	1.98
		Winter	2.19	1.25
K	meq/100g	Summer	0.26	0.19
		Winter	0.64	0.20
Ca	meq/100g	Summer	10.30	5.30
		Winter	7.16	3.64
P	μg/g	Summer	9.76	5.16
		Winter	11.23	7.52
Mg	meq/100g	Summer	2.13	1.65
		Winter	1.27	1.42
Mn	μg/g	Summer	146.55	25.83
		Winter	115.46	22.46
Fe	μg/g	Summer	133.39	25.49
		Winter	104.79	23.12

#### 3.2.3 Soil fertility analysis of coal water treated soil

##### 3.2.3.1 pH levels and total organic matter

An analysis was conducted on coal water treated soil (CTS-8) to determine soil pH and total organic matter (OM) content. During the summer season, the soil pH value was 6.30, which classified it as slightly acidic according to the Bangladesh Agricultural Research Council (BARC). However, in the winter season, the pH value increased to 7.10, indicating a shift to neutral conditions. This change from slightly acidic to neutral pH suggests an improvement in soil reaction, potentially enhancing nutrient availability and overall soil health. In terms of organic matter content, the soil exhibited very high levels in both seasons. In the summer, the OM content was 7.91 %, which is considered very high according to BARC standards. In the winter, the OM content slightly decreased to 7.17 % but still fell within the very high category. The consistently high organic matter content throughout the seasons indicates that the soil is rich in organic materials, which are crucial for maintaining soil structure, moisture retention, and nutrient supply [58]. The seasonal analysis revealed a slight decrease in organic matter during the winter, but the pH level showed a significant shift towards neutrality. These changes highlight the dynamic nature of soil properties and emphasize the importance of seasonal monitoring for optimal soil management. With its high organic matter levels and favorable pH conditions, CTS-8 soil farmed with water near mine drainage appears to positively impact soil fertility, making it suitable for agricultural use throughout the year.

##### 3.2.3.2 Analysis of nutrients

The analysis of nutrient levels in soil treated with coal water (CTS-8) provides valuable insights into soil fertility and seasonal variations. The concentrations of essential nutrients were evaluated against the standard nutrient level chart provided by the Bangladesh Agricultural Research Council (BARC). Both zinc (Zn) and copper (Cu) levels were found to be “very high” in both the summer and winter seasons, indicating an abundance of these micronutrients in the soil. While these elements are necessary for plant growth, excessive levels may pose risks of phytotoxicity and potential environmental concerns. Potassium (K) levels exhibited seasonal fluctuations, with an “optimum” concentration observed during the summer season and a “very high” level recorded in the winter. This variation may be attributed to changes in soil moisture content, temperature, or other environmental factors that influence potassium availability.

Calcium (Ca) and magnesium (Mg) displayed contrasting seasonal patterns. The concentration of Ca was found to be “very high” during the summer but decreased to “high” levels in the winter, whereas Mg levels transitioned from “very high” in the summer to “optimum” in the winter. These variations may be attributed to differences in soil pH, cation exchange capacity, or other soil physicochemical properties that impact the availability and retention of these macronutrients. Phosphorus (P) levels remained consistently “low” throughout both seasons, indicating a potential deficiency or limited availability of this essential macronutrient in the soil. This discovery could have implications for plant growth and productivity, therefore necessitating appropriate management strategies to enhance phosphorus availability. The concentrations of manganese (Mn) and iron (Fe) were classified as “very high” in both seasons, suggesting an abundance of these micronutrients in the soil. Although crucial for plant growth, excessive levels of these elements may also pose potential risks and require meticulous monitoring. The observed seasonal variations in nutrient levels underscore the dynamic nature of soil properties and the impact of environmental factors on nutrient availability and cycling. These findings emphasize the significance of monitoring soil nutrient status and implementing appropriate management practices to ensure optimal soil fertility and sustainable agricultural production.

## 4 Conclusions and recommendations

This study presents a comparative analysis of water and soil quality parameters in the Barapukuria coal mining area, focusing on the seasonal variations in environmental impacts during the summer and winter seasons. By examining water quality for various uses (irrigation, disposal, drinking) and soil quality (heavy metal contamination, fertility), the research provides a comprehensive understanding of the environmental status of the mining area during these distinct seasons. The analysis of surface water quality indicates that it is generally suitable for irrigation purposes. However, treatment is necessary to address elevated levels of turbidity and COD. In contrast, the wastewater exhibited seasonal fluctuations, with turbidity, TSS, and COD levels exceeding standard guidelines, particularly during the winter season. These findings underscore the critical need for proper treatment of wastewater before disposal to mitigate potential environmental risks. The evaluation of drinking water quality using the WQI revealed that the water is unsuitable for consumption year-round, with higher contamination levels observed during the summer compared to the winter season. Although deep tubewell water demonstrated slightly better quality, treatment measures remain essential. These results emphasize the importance of implementing comprehensive water treatment strategies and continuous monitoring programs to protect public health from the seasonal impacts of mining activities. The assessment of heavy metal contamination in surface soil adjacent to mine drainage, based on the Geo-accumulation Index, indicated minimal to moderate contamination by cadmium (Cd), while other metals (Cu, Zn, Pb, and Fe) were found at uncontaminated levels. Seasonal variations showed a slight reduction in Cd contamination during the winter season. Additionally, a comparison between coal water-treated soil (CTS-8) and normal field soil (NFS-9) demonstrated significant improvements in soil quality in CTS-8, characterized by increased organic matter content, elevated micronutrient levels, and favorable pH changes. Seasonal variations also revealed higher nutrient concentrations during the winter season, suggesting enhanced soil fertility. However, this study has limitations, primarily due to the limited number of samples, which affects the depth of the analysis. Future research should include a larger sample size, examine additional seasons, incorporate statistical analyses, and employ advanced techniques such as Inductively Coupled Plasma Mass Spectrometry (ICP-MS) for metal quantification to achieve a more comprehensive understanding of environmental impacts. Expanding the scope and temporal coverage will enable more precise insights and facilitate the development of effective mitigation strategies for mining areas. While the study provides valuable insights into the presence of coliform bacteria in drinking water, it is important to acknowledge that the samples were not collected under sterile conditions. The lack of strict aseptic techniques during sampling could introduce microbial contamination, potentially influencing the accuracy of the results. Future research should address this limitation by employing sterilized bottles and adhering to strict aseptic techniques during sample collection to ensure sample integrity. The findings of this study underscore the significance of considering seasonal dynamics in soil and water management practices in proximity to mining areas. Continuous monitoring and the implementation of appropriate management practices are essential to ensure the long-term environmental sustainability of the mining area and mitigate potential adverse impacts on soil, water, and public health.

## CRediT authorship contribution statement

**Md Asif All Azad:** Writing – review & editing, Writing – original draft, Visualization, Methodology, Investigation, Formal analysis, Conceptualization. **Abu Bakker Chiddiq:** Writing – review & editing. **Md Rubel Miah:** Writing – review & editing. **Md Hafijur Rahman Sabbir:** Writing – review & editing.

## Data availability statement

The authors confirm that the data supporting the findings of this study are available within the article and its supplementary materials.

## Declaration of generative AI and AI-assisted technologies in the writing process

During the preparation of this work, the author(s) used Wordtune and Grammarly to improve readability, grammar, and language clarity. After using these tools, the author(s) reviewed and edited the content as needed and take(s) full responsibility for the content of the publication.

## Funding

This research did not receive any specific grant from funding agencies in the public, commercial, or not-for-profit sectors.

## Declaration of competing interest

The authors declare that they have no known competing financial interests or personal relationships that could have appeared to influence the work reported in this paper.
