# Supplementary material for: Temporal assessment of water and soil quality near Barapukuria coal mine, Bangladesh
Source: Heliyon. 2024 Nov 27;10(23):e40722. doi: 10.1016/j.heliyon.2024.e40722 (PMC11647810; doi:10.1016/j.heliyon.2024.e40722)
Supplement: Multimedia component 1 [file mmc1.docx]

**Appendix A**

**A1.** Water quality parameter values in surface water

| **Parameter** | **Unit** | **Season** | **Surface water (SW-1)** | **Surface water (SW-2)** |
| --- | --- | --- | --- | --- |
| pH | - | Summer | 7.40 | 7.10 |
|  |  | Winter | 7.50 | 7.60 |
| Turbidity | NTU | Summer | 39.10 | 14.10 |
|  |  | Winter | 15.10 | 10.00 |
| Color | Pt-Co | Summer | 217.00 | 76.00 |
|  |  | Winter | 71.00 | 470.00 |
| Hardness | mg/L | Summer | 254.65 | 231.50 |
|  |  | Winter | 199.09 | 134.27 |
| TS | mg/L | Summer | 232.00 | 350.00 |
|  |  | Winter | 115.00 | 220.00 |
| TDS | mg/L | Summer | 110.00 | 180.00 |
|  |  | Winter | 50.00 | 100.00 |
| TSS | mg/L | Summer | 122.00 | 170.00 |
|  |  | Winter | 65.00 | 120.00 |
| EC | µS/cm | Summer | 314.00 | 321.00 |
|  |  | Winter | 360.00 | 341.00 |
| DO | mg/L | Summer | 6.20 | 7.15 |
|  |  | Winter | 9.20 | 8.60 |
| BOD_5_ | mg/L | Summer | 1.43 | 0.72 |
|  |  | Winter | 1.70 | 1.50 |
| COD | mg/L | Summer | 199.94 | 629.57 |
|  |  | Winter | 233.79 | 473.34 |
| NO_3_^-^ | mg/L | Summer | 0.88 | 0.60 |
|  |  | Winter | 0.00 | 0.00 |
| SO_4_^2-^ | mg/L | Summer | 15.00 | 26.00 |
|  |  | Winter | 11.00 | 17.00 |
| Na^+^ | mg/L | Summer | 25.92 | 14.58 |
|  |  | Winter | 16.20 | 9.72 |
| Cl^-^ | mg/L | Summer | 40.00 | 22.50 |
|  |  | Winter | 25.00 | 15.00 |
| PO_4_^3-^ | mg/L | Summer | 0.032 | 0.053 |
|  |  | Winter | 0.013 | 0.011 |

**A2.** Water quality parameter values in wastewater

| **Parameters** | **Unit** | **Season** | **Wastewater (MDW-3)** | **Wastewater (MDW-4)** |
| --- | --- | --- | --- | --- |
| pH | - | Summer | 7.10 | 7.00 |
|  |  | Winter | 7.30 | 7.20 |
| Turbidity | NTU | Summer | 24.60 | 27.10 |
|  |  | Winter | 55.10 | 29.80 |
| Color | Pt-Co | Summer | 162.00 | 195.00 |
|  |  | Winter | 267.00 | 129.00 |
| Hardness | mg/L | Summer | 263.91 | 287.06 |
|  |  | Winter | 180.57 | 138.90 |
| TS | mg/L | Summer | 193.00 | 202.00 |
|  |  | Winter | 210.00 | 225.00 |
| TDS | mg/L | Summer | 170.00 | 182.00 |
|  |  | Winter | 90.00 | 130.00 |
| TSS | mg/L | Summer | 23.00 | 20.00 |
|  |  | Winter | 120.00 | 95.00 |
| DO | mg/L | Summer | 7.31 | 5.88 |
|  |  | Winter | 9.40 | 9.30 |
| BOD_5_ | mg/L | Summer | 1.49 | 1.83 |
|  |  | Winter | 2.30 | 2.20 |
| COD | mg/L | Summer | 462.93 | 663.42 |
|  |  | Winter | 332.74 | 397.83 |
| EC | µS/cm | Summer | 314.00 | 316.00 |
|  |  | Winter | 317.00 | 329.00 |
| Fe | mg/L | Summer | 0.91 | 0.81 |
|  |  | Winter | 0.37 | 0.66 |
| Mn | mg/L | Summer | 0.70 | 0.20 |
|  |  | Winter | 0.20 | 0.10 |
| Cl^-^ | mg/L | Summer | 62.50 | 35.00 |
|  |  | Winter | 27.50 | 30.00 |
| As | µg/L | Summer | BDL* | BDL* |
|  |  | Winter | BDL* | BDL* |

*BDL: Below Detection Limit

**Appendix B**

**B1.** Classification of soil pH and organic matter according to Bangladesh Agricultural Research Council (BARC) [1]

| **Standard of soil pH** | | **Standard of soil organic matter (%)** | |
| --- | --- | --- | --- |
| Value | Soil Reaction Class | Value | Status |
| <4.5 | Very strongly acidic | <1.00 | Very low |
| 4.5-5.5 | Strongly acidic | 1.00-1.70 | Low |
| 5.6-6.5 | Slightly acidic | 1.71-3.40 | Medium |
| 6.6-7.3 | Neutral | 3.41-5.50 | High |
| 7.4-8.4 | Slightly alkaline | >5.50 | Very high |
| 8.5-9.0 | Strongly alkaline | - | - |
| >9.0 | Very strongly alkaline | - | - |

**B2.** Nutrients level chart of soil according to Bangladesh Agricultural Research Council [1]

|  | **Nutrients status** | | | | | |
| --- | --- | --- | --- | --- | --- | --- |
| **Nutrient** | **Very low** | **Low** | **Medium** | **Optimum** | **High** | **Very high** |
| N (%) | <0.090 | 0.091 - 0.18 | 0.081 - 0.270 | 0.271 - 0.36 | 0.361 - 0.450 | >0.450 |
| P (µg/g) | <6.000 | 6.100 - 12.00 | 12.00 - 18.000 | 18.000 - 24.00 | 24.00 - 30.000 | >30.000 |
| Zn (µg/g) | <0.450 | 0.451 - 0.90 | 0.910 - 1.350 | 1.351 - 1.80 | 1.810 - 2.250 | >2.250 |
| Fe (µg/g) | <3.000 | 3.100 - 6.00 | 6.100 - 9.000 | 9.100 - 12.00 | 12.00 - 15.000 | >15.000 |
| Mn (µg/g) | <0.750 | 0.760 - 1.50 | 1.510 - 2.250 | 2.260 - 3.00 | 3.100 - 3.750 | >3.750 |
| B (µg/g) | <0.150 | 0.151 - 0.30 | 0.310 - 0.450 | 0.451 - 0.600 | 0.610 - 0.750 | >0.750 |
| K (meq/100g) | <0.075 | 0.076 - 0.15 | 0.151 - 0.225 | 0.226 - 0.300 | 0.310 - 0.375 | >0.375 |
| Ca (meq/100g) | <1.500 | 1.510 - 3.00 | 3.010 - 4.500 | 4.510 - 6.00 | 6.010 - 7.500 | >7.500 |
| Mg (meq/100g) | <0.375 | 0.376 - 0.75 | 0.751 - 1.125 | 1.126 - 1.50 | 1.510 - 1.875 | >1.875 |
| Cu (µg/g) | <0.15 | 0.15 - 0.30 | 0.31 - 0.45 | 0.45 - 0.60 | 0.61 - 0.75 | >0.75 |

**Reference**:

[1] M. Islam, T. Nusrat, M. Jamil, F. Yesmin, M. Kabir, R. Rimi, Investigation of soil properties and nutrients in agricultural practiced land in Tangail, Bangladesh, Int. J. Agril. Res. Innov. & Tech. 10 (2021) 84–90. https://doi.org/10.3329/ijarit.v10i2.51581.

**Appendix C**

**pH Meter**

- Model: PH-5011
- Manufacturer: Ezodo
- Measurement Accuracy: ±0.2 pH

**Turbidity Meter**

- Model: 2100Q Portable Turbidimeter (EPA)
- Manufacturer: Hach
- Measurement Range: 0 to 1000 NTU (Nephelometric Turbidity Units)
- Measurement Accuracy: ±2% of the reading plus stray light

**Spectrophotometer**

- Model: DR3900 Laboratory VIS Spectrophotometer
- Manufacturer: Hach
- Photometric Accuracy: ±5 mAbs @ 0.0 - 0.5 Abs, ±1% at 0.50 - 2.0 Abs
- Wavelength Accuracy: ±1.5 nm (wavelength range 340 - 900 nm)

**Glass Burette**

- Material: 50 ml borosilicate ASTM burette
- Stopcock: Teflon stopcock, single straight bore
- Class: B
- Graduations: 0.1 ml ceramic graduations
- Tolerance: ±0.10 ml
- Material: High-quality 3.3 borosilicate glass
- Standards: Complies with DIN ISO 385 and ASTM E1272

**Drying Oven**

- Model: SH-DO-100FG
- Manufacturer: SH Scientific Korea
- Air Circulation Type: Forced
- Temperature Range: Ambient +10℃ to 250℃ (Ambient +18℉ to 482℉)
- Time Range: 00.00 to 99HR 59MIN (MIN SEC) Selectable Digital Counter Timer
- Controller: Microprocess PID Membrane Touch Digital controller

**Multimeter**

- Model: WA-2015
- Manufacturer: Lutron

**Dissolved Oxygen Meter**

- Model: HQ1130 DO/1 Channel
- Manufacturer: Hach
- DO Measurement Range: 0.1 - 20.0 mg/L (ppm), 1 - 200% saturation

**Incubator**

- Model: Low-Profile Culture Model 153
- Manufacturer: Hach
- Measurement Accuracy: ±0.1 °C at 37 °C
- Temperature Uniformity: ±0.25 °C at 37 °C
